# Mowat-Wilson syndrome: growth charts

**DOI:** 10.1186/s13023-020-01418-4

**Published:** 2020-06-15

**Authors:** Ivan Ivanovski, Olivera Djuric, Serena Broccoli, Stefano Giuseppe Caraffi, Patrizia Accorsi, Margaret P. Adam, Kristina Avela, Magdalena Badura-Stronka, Allan Bayat, Jill Clayton-Smith, Isabella Cocco, Duccio Maria Cordelli, Goran Cuturilo, Veronica Di Pisa, Juliette Dupont Garcia, Roberto Gastaldi, Lucio Giordano, Andrea Guala, Christina Hoei-Hansen, Mie Inaba, Alessandro Iodice, Jens Erik Klint Nielsen, Vladimir Kuburovic, Brissia Lazalde-Medina, Baris Malbora, Seiji Mizuno, Oana Moldovan, Rikke S. Møller, Petra Muschke, Valeria Otelli, Chiara Pantaleoni, Carmelo Piscopo, Maria Luisa Poch-Olive, Igor Prpic, Purificación Marín Reina, Federico Raviglione, Emilia Ricci, Emanuela Scarano, Graziella Simonte, Robert Smigiel, George Tanteles, Luigi Tarani, Aurelien Trimouille, Elvis Terci Valera, Samantha Schrier Vergano, Karin Writzl, Bert Callewaert, Salvatore Savasta, Maria Elisabeth Street, Lorenzo Iughetti, Sergio Bernasconi, Paolo Giorgi Rossi, Livia Garavelli

**Affiliations:** 1 Medical Genetics Unit, Department of Mother and Child, Azienda Unità Sanitaria Locale - IRCCS di Reggio Emilia, Viale Risorgimento, 80 42123 Reggio Emilia, Italy; 2 Department of Surgical, Medical, Dental and Morphological Sciences with interest in Transplant, Oncology and Regenerative Medicine, University of Modena and Reggio Emilia, Modena, Italy; 3 Institut für Medizinische Genetik, Universität Zürich, Zürich, Switzerland; 4 Epidemiology Unit, Azienda Unità Sanitaria Locale - IRCCS di Reggio Emilia, Reggio Emilia, Italy; 5 Center for Environmental, Nutritional and Genetic Epidemiology (CREAGEN), Section of Public Health, Department of Biomedical, Metabolic and Neural Sciences, University of Modena and Reggio Emilia, Modena, Italy; 6 Neuropsychiatric Department, Spedali Civili Brescia, Brescia, Italy; 7 Division of Genetic Medicine, University of Washington School of Medicine, Seattle, Washington, USA; 8 Department of Clinical Genetics, Helsinki University Hospital, Helsinki, Finland; 9 Chair and Department of Medical Genetics, Poznan University of Medical Sciences, Poznań, Poland; 10 Institute for Regional Health Service, University of Southern Denmark, Odense, Denmark; 11 Department of Epilepsy Genetics and Personalized Medicine, Danish Epilepsy Centre Dianalund, Dianalund, Denmark; 12 Division of Evolution and Genomic Sciences, School of Biological Sciences, Faculty of Biology, Medicine and Health, University of Manchester, Manchester, UK; 13 Manchester Centre for Genomic Medicine, St Mary’s Hospital, Manchester University NHS Foundation Trust, Health Innovation Manchester, Manchester, UK; 14 Child Neurology and Psychiatry Unit, Pediatric Department, St. Orsola-Malpighi Hospital, University of Bologna, Bologna, Italy; 15 Faculty of Medicine, University of Belgrade, Belgrade, Serbia; 16 Department of Medical Genetics, University Children’s Hospital, Belgrade, Serbia; 17 Serviço de Genética Médica, Departamento de Pediatria, Hospital de Santa Maria, Centro Hospitalar Lisboa Norte, Centro Académico de Medicina de Lisboa, Lisbon, Portugal; 18 UOC Clinica Pediatrica, Istituto Giannina Gaslini, Genoa, Italy; 19 SOC Pediatria, Ospedale Castelli, Verbania, Italy; 20 Department of Paediatrics, Copenhagen University Hospital, Rigshospitalet, Denmark; 21 Department of Pediatrics, Central Hospital, Aichi Human Service Center, Kasugai, Japan; 22 Child Neurology and Psychiatry Unit, Azienda Unità Sanitaria Locale - IRCCS di Reggio Emilia, Reggio Emilia, Italy; 23 Department of Pediatrics, Zealand University Hospital Roskilde, Roskilde, Denmark; 24 Department of Cardiology, Mother and Child Health Care Institute, Belgrade, Serbia; 25 Skånes universitet sjukhus, Barnkliniken, Lund, Sweden; 26 Biomedical Research Unit, Mexican Institute of Social Security, Durango, Mexico; 27 Department of Pediatric Hematology & Oncology, Tepecik Training and Research Hospital, Izmir, Turkey; 28 Danish Epilepsy Centre, Dianalund, Denmark; 29 Institute for Regional Health Services, University of Southern Denmark, Odense, Denmark; 30 Institute for Human Genetics, University Hospital Magdeburg, Magdeburg, Germany; 31 ATS Bergamo, Brembana Valley district, Bergamo, Italy; 32 Developmental Neurology Unit, Fondazione IRCCS Istituto Neurologico Carlo Besta, Milan, Italy; 33 U.O.S.C. Medical Genetics, A.O.R.N. “A. Cardarelli”, Naples, Italy; 34 Department of Pediatrics, H. San Pedro, La Rioja, Logrono, Spain; 35 Department of Pediatrics–Child Neurology Service, University Hospital Rijeka, Medical Faculty, University of Rijeka, Rijeka, Croatia; 36 Dismorphology and Reproductive Genetics, Neonatal Research Group, Health Research Institute Hospital La Fe, University & Polytechnic Hospital La Fe, Valencia, Spain; 37 Child Neuropsychiatry Unit, U.O.N.P.I.A ASST–Rhodense, Rho, Milan, Italy; 38 Unit of Pediatrics, Department of Medical and Surgical Sciences, St. Orsola-Malpighi Hospital, University of Bologna, Bologna, Italy; 39 Department of Pediatrics and Medical Sciences, “Vittorio Emanuele” Hospital, University of Catania, Catania, Italy; 40 Department of Pediatrics, Division Pediatric Propedeutics and Rare Disorders, Wroclaw Medical University, Wroclaw, Poland; 41 Clinical Genetics Clinic, Cyprus Institute of Neurology and Genetics, Nicosia, Cyprus; 42 Department of Pediatrics, University “La Sapienza,”, Rome, Italy; 43 CHU de Bordeaux, Service de Génétique Médicale, Bordeaux, France; 44 INSERM U1211, Univ. Bordeaux, Bordeaux, France; 45 Department of Pediatrics, Ribeirão Preto Medical School, University of São Paulo, São Paulo, Brazil; 46 Department of Pediatrics, Eastern Virginia Medical School, Norfolk, Virginia, USA; 47 Division of Medical Genetics and Metabolism, Children’s Hospital of The King’s Daughters, Norfolk, Virginia, USA; 48 Clinical Institute of Medical Genetics, University Medical Centre Ljubljana, Ljubljana, Slovenia; 49 Center for Medical Genetics, Ghent University Hospital, Ghent, Belgium; 50 Department of Biomolecular Medicine, Ghent University, Ghent, Belgium; 51 Pediatric Clinic, IRCCS Policlinico “S. Matteo” Foundation, University of Pavia, Pavia, Italy; 52 Division of Pediatric Endocrinology and Diabetology, Department of Mother and Child, Azienda Unità Sanitaria Locale - IRCCS di Reggio Emilia, Reggio Emilia, Italy; 53 Post-graduate School of Pediatrics, University of Modena and Reggio Emilia, Modena, Italy; 54 Department of Medical and Surgical Sciences of Mother, Children and Adults, Pediatric Unit, University of Modena and Reggio Emilia, Modena, Italy; 55 Microbiome Research Hub, University of Parma, Parma, Italy

**Keywords:** Mowat-Wilson syndrome, *ZEB2*, Growth charts, Weight, Length, Height, Head circumference, Body mass index, BMI

## Abstract

**Background:**

Mowat–Wilson syndrome (MWS; OMIM #235730) is a genetic condition caused by heterozygous mutations or deletions of the *ZEB2* gene. It is characterized by moderate-severe intellectual disability, epilepsy, Hirschsprung disease and multiple organ malformations of which congenital heart defects and urogenital anomalies are the most frequent ones. To date, a clear description of the physical development of MWS patients does not exist. The aim of this study is to provide up-to-date growth charts specific for infants and children with MWS. Charts for males and females aged from 0 to 16 years were generated using a total of 2865 measurements from 99 MWS patients of different ancestries. All data were collected through extensive collaborations with the Italian MWS association (AIMW) and the MWS Foundation. The GAMLSS package for the R statistical computing software was used to model the growth charts. Height, weight, body mass index (BMI) and head circumference were compared to those from standard international growth charts for healthy children.

**Results:**

In newborns, weight and length were distributed as in the general population, while head circumference was slightly smaller, with an average below the 30th centile. Up to the age of 7 years, weight and height distribution was shifted to slightly lower values than in the general population; after that, the difference increased further, with 50% of the affected children below the 5th centile of the general population. BMI distribution was similar to that of non-affected children until the age of 7 years, at which point values in MWS children increased with a less steep slope, particularly in males. Microcephaly was sometimes present at birth, but in most cases it developed gradually during infancy; many children had a small head circumference, between the 3rd and the 10th centile, rather than being truly microcephalic (at least 2 SD below the mean). Most patients were of slender build.

**Conclusions:**

These charts contribute to the understanding of the natural history of MWS and should assist pediatricians and other caregivers in providing optimal care to MWS individuals who show problems related to physical growth. This is the first study on growth in patients with MWS.

## Background

Mowat-Wilson syndrome (MWS; OMIM #235730) is a rare autosomal dominant disorder caused by haploinsufficency in the *ZEB2* gene located on chromosome 2. It is characterized by distinctive facial features, moderate-to-severe intellectual disability, epilepsy, Hirschsprung disease and multiple congenital anomalies, including genital anomalies, congenital heart defects, agenesis of the corpus callosum and eye defects. Since the first report in 1998, more than 350 individuals have been described in the literature [1–8].

The incidence of MWS is estimated to be 1:50.000–70.000 live births [9].

Several studies have reported the presence of short stature in MWS patients. In a recent article [7], which included the clinical data of 87 patients with MWS, we demonstrated that basic growth parameters - weight, length or height, body mass index (BMI), head circumference - are often normal at birth, but have a tendency to descend well below the normal range in childhood and later on. These results urged us to extend our study and construct growth charts specific for MWS.

Growth charts specific for a disease are fundamental instruments in order to monitor growth and observe deviations from normal patterns [10–20]. They allow clinicians to advise parents concerning growth expectations for their children, and they can be very useful even in a group of patients for whom growth anomalies are not the most severe problem. Moreover, these charts describe the natural history of the disease.

The aim of this study was to provide a growth reference for MWS patients. We collected the measurements of 99 MWS patients from 20 countries and developed specific growth charts that describe the natural history of height, weight, BMI and head circumference in MWS.

## Methods

### Study population

Growth data of MWS patients previously published by our workgroup were incorporated into this study [7]. An invitation to participate in the study, explaining its purpose and design, was sent to all our colleagues who had a previous history of publications on MWS. Furthermore, the Italian Mowat-Wilson Association (AIMW) and the MWS Foundation forwarded the invitation to their members. To those who agreed to participate, we sent a specially designed questionnaire where they could insert the length/height, weight and head circumference measurements of patients at different ages. The collected data were reviewed and assembled in a password-protected database. Whenever we detected inconsistencies, probable errors or missing data, we contacted the family/referring doctor for corrections and further information, if needed. Only individuals with both clinical and molecular confirmation of the disease were included in the study. Two patients were excluded since the causative *ZEB2* variant could not be found despite molecular testing.

A total of 20 nationalities were represented in this study: 38 Italian patients, 16 from the USA, 7 from Poland, 6 from Japan, 5 from Denmark, 4 from Spain, 4 from the UK, 3 from Portugal, 3 from Finland, 2 from Croatia, 2 from Serbia, and 1 each from Albania, Brazil, Chile, Cyprus, France, Germany, Mexico, Turkey and Ukraine. None of the affected individuals originated from a consanguineous family. One Italian family comprised two affected siblings. None of the patients had ever been treated with growth hormone.

A total of 99 patients were included in the study, 53 females and 46 males, born between 1986 and 2016. A total of 2865 measurements were available: 1220 data points from 46 males and 1645 data points from 53 females. These patients had a mean age of 12 years (range 4–20 years) in males and 13 years (range 3–23 years) in females at the time of assessment.

A total of 1013 height measurements (573 for females, 440 for males), 1110 weight measurements (631 for females, 479 for males), and 742 head circumference measurements (441 for females, 301 for males) were available. We gathered measurements from at least 2 time points in 97 patients, and from more than 4 time points in 89 patients. For each patient we had an average of 10.39 (SD 6.52) height measurements, 11.37 (SD 6.77) weight measurements, and 7.64 (SD 5.76) head circumference measurements (Additional file 1 Table 1).

Due to limited data availability, no growth curves could be generated for individuals with MWS past the age of 16 years.

Data from 1986 to 2019 were included. Height, weight and head circumference parameters were analyzed from birth until 23 years of age, both for males and females. BMI was calculated from these data, using the formula BMI = mass(kg)/(height(m))^2^.

Distribution centiles for weight, length and head circumference at birth were computed including preterm and at term newborns, i.e. from 32 to 42 gestational weeks (mean 36.5), and presented separately for males and females.

The data after birth were divided into different age and sex groups, with 1-month intervals during the first year of life, 3-month intervals during the 2nd and 3rd years of life, and 6-month intervals between the 4th and 23rd years of life.

Our growth charts were compared with the reference percentiles for anthropometric measurements in healthy children provided by the Centers for Disease Control and Prevention (CDC), with a few exceptions: for neonatal anthropometric assessment we referred to the Italian Neonatal Study (INeS) growth charts, data on head circumference in males and females aged between 3 and 16 years were compared with the Tanner reference percentiles, and standard deviations of the head circumference in males and females aged between birth and 18 years were compared with the Nellhaus reference standard deviations [21–28].

### Statistical analysis

The growth charts were developed using the Generalized Additive Models for Location, Scale and Shape (GAMLSS) [29, 30] package for the R statistical computing software [31].

Centile estimation of anthropometric data *Y* (weight, length, BMI and head circumference) for any given age was:

$$ {\displaystyle \begin{array}{l}\mathrm{Y}\sim \mathrm{D}\ \left(\upmu, \updelta, \upnu, \uptau \right)\\ {}{\mathrm{g}}_1\left(\upmu \right)={\mathrm{h}}_1\left(\mathrm{x}\right)\\ {}{\mathrm{g}}_2\left(\updelta \right)={\mathrm{h}}_2\left(\mathrm{x}\right)\\ {}{\mathrm{g}}_3\left(\upnu \right)={\mathrm{h}}_3\left(\mathrm{x}\right)\\ {}{\mathrm{g}}_4\left(\uptau \right)={\mathrm{h}}_4\left(\mathrm{x}\right)\\ {}\mathrm{x}={age}^{\xi}\end{array}} $$

where the distribution *D* represents the best general probability (density) function according to Akaike Information Criterion (AIC) and *μ* the first parameter of the distribution (usually location)*, δ* the second parameter of the distribution (usually scale, coefficient of variation)*, ν* the third parameter of the distribution (usually shape, e.g. skewness)*, τ* the fourth parameter of the distribution (usually shape, e.g. kurtosis).

The *g()* functions represent appropriate link functions, *h()* are non-parametric smoothing functions and *ξ* is a power transformation of age (Additional file 2 Table 2).

The more traditional LMS method developed by Cole and Green [32] can be fitted within this framework by assuming that the response variable has a Box-Cox Cole and Green distribution [33, 34]. Additional file 2 Table 2 describes the model specifications by anthropometric measure and sex. The Q test was conducted to evaluate the fit of the model [35].

The estimated centiles by age and sex were converted back to Excel to create charts for each anthropometric variable, including the reference CDC growth charts for healthy children.

## Results

### Length, weight and head circumference at birth

Information on weight at birth was available for 47 females and 35 males. Information on length at birth was available for 47 females and 31 males. Information on head circumference at birth was available for 40 females and 26 males.

The average gestational age was 39 weeks, with a range between 32 and 42. Gestational age in MWS was completely comparable to that of the general population.

Weights at birth were found to be within the normal range in both males and females. The average weight in males was 3288 g, with a range between 1850 g and 4500 g; the average weight in females was 3340 g, with a range between 1620 g and 4060 g. The birth weight of all males was between the 2nd and 100th centiles for gestational age (mean 51st centile); the females’ birth weight was between the 3rd and 100th centiles (mean 54th centile).

Length at birth was within the normal range for the vast majority of patients, both males and females. The average birth length in males measured 50 cm, with a range between 43 and 56 cm (range 2nd to 99th centile, mean 44th centile). The average birth length in females was 49.1 cm, with a range between 41 and 54 cm (range < 1st to 100th centile, mean 53rd centile).

Despite the fact that in both sexes the average head circumference at birth was within the normal range, most patients were under the 50th centile, with mean values at the 27th centile for males and 31st for females. In males, the mean was 33.2 cm, with a range between 28.5 and 36 cm (<1st to 96th centile); in females, the mean was 33.7 cm, with a range between 28.5 and 36 cm (<1st to 100th centile).

### Length and height

The mean number of longitudinal measurements per patient in males was 9.56 (SD 5.51). For 45 patients we had at least 2 measurements, and for 40 patients we gathered more than 4 measurements.

The mean number of longitudinal measurements per patient in females was 11.00 (SD 7.11). For 53 patients we had at least 2 measurements, and for 48 patients we gathered more than 4 measurements.

**Fig. 1:**
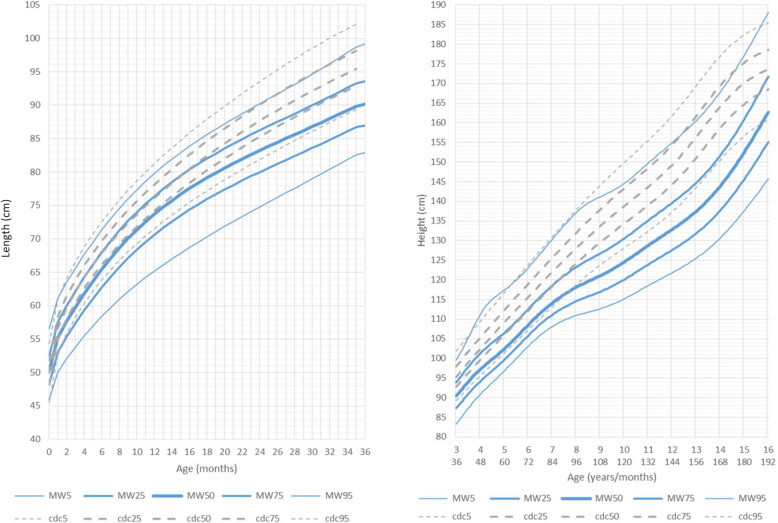
Constructed length and height growth charts for MWS patients in relation to the CDC reference charts (black) for males (blue)

Up to the age of 2 months, the length of both males and females was within, or just below, the normal range. Subsequently, height in males began to increase more slowly. A significant reduction in the slope of the growth curves was noted after 7 years of age, as the height of boys affected by MWS became even more distant from the reference curves of the general population (Fig. 1 and Additional file 3 Fig. RD1).

Mean height for male patients at the age of 9 years was 121 cm (<3rd percentile, mean reference group at 50th percentile: 133 cm) and for female patients was 123.8 cm (3rd-5th percentile, mean reference group at 50th percentile: 130 cm).

**Fig. 2:**
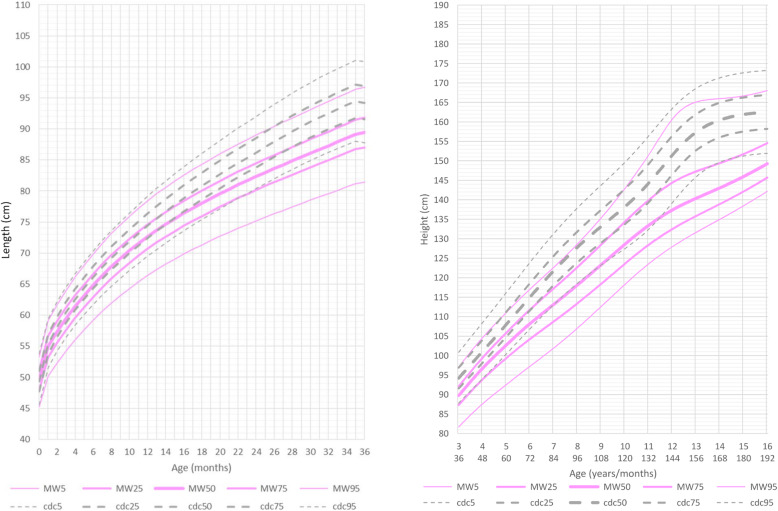
Constructed length and height growth charts for MWS patients in relation to the CDC reference charts (black) for females (pink)

In females, from 2 months of age, height had a slower increase as in males, and subsequently diverged even further from the reference curves of the general population. A significant reduction in the slope of the growth charts was noted after 11 years of age, much later than in males. At this age, females affected by MWS became noticeably distant from the reference curves of the general population, but were slightly less distant than males (Fig. 2 and Additional file 4 Fig. RD2).

We were not able to extend the growth charts over the age of 16 years due to the limited availability of measurements after this age. Furthermore, it is not yet possible to calculate the final height because MWS has only relatively recently been described and the data on adolescent and young adult patients is still very limited.

### Weight and body mass index

The mean number of weight measurements per patient in males was 10.41 (SD 5.67). For 45 patients we had at least 2 measurements and for 41 patients we gathered more than 4 measurements.

The mean number of weight measurements per patient in females was 12.07 (SD 7.41). For 52 patients we had at least 2 measurements and for 50 patients we collected more than 4 measurements.

At age 0 to 36 months, compared to the reference group, the growth curves for weight were slightly lower than normal in both genders. Mean weight for male patients at age 5 years and 6 months was 16.800 kg (10th percentile, mean reference group at 50th percentile: 19.500 kg) and for female patients was 15.400 kg (10th percentile, mean reference group at 50th percentile: 19 kg).

**Fig. 3:**
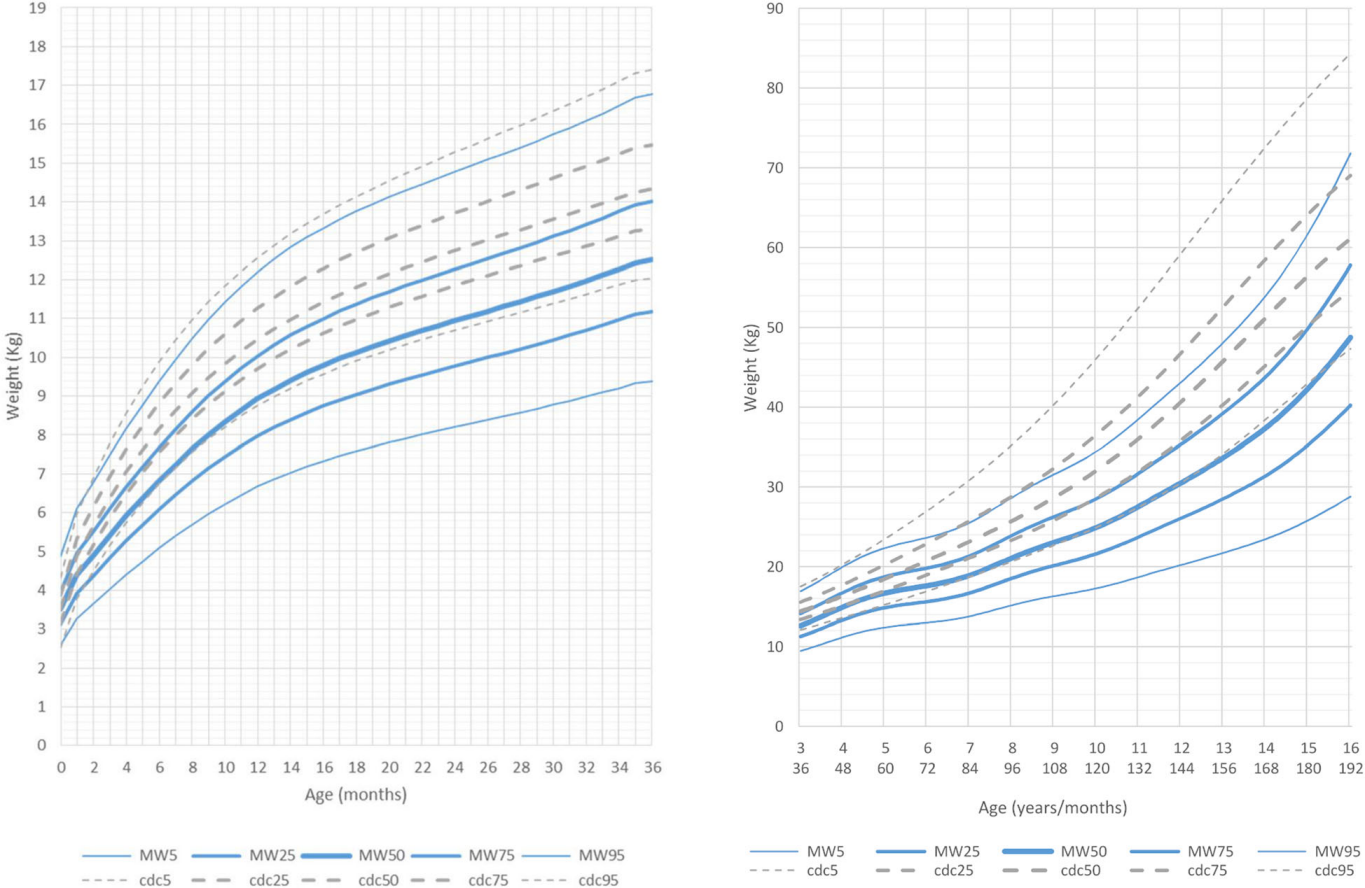
Constructed weight growth charts for MWS patients in relation to the CDC reference charts (black) for males (blue)

**Fig. 4:**
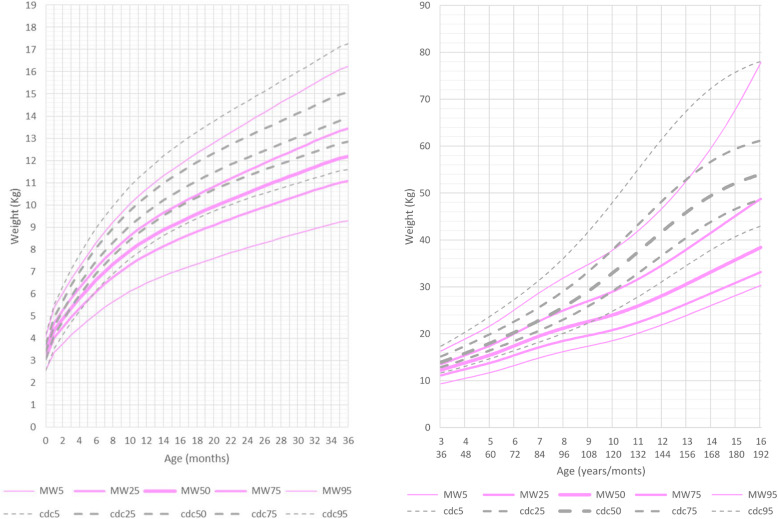
Constructed weight growth charts for MWS patients in relation to the CDC reference charts (black) for females (pink)

After this age the growth charts flatten out considerably when compared to the normal reference group (Figs. 3 and 4, Additional file 5 Fig.RD3, Additional file 6 Fig. RD4).

At the age of 16 years both male and female patients were considerably lighter (males: mean 48 kg 5th percentile, mean reference group at 50th percentile: 61 Kg; females: mean 39 kg <3rd percentile, mean reference group at 50th percentile: 54 Kg). Most patients were of slender build, with normal body proportions.

**Fig. 5:**
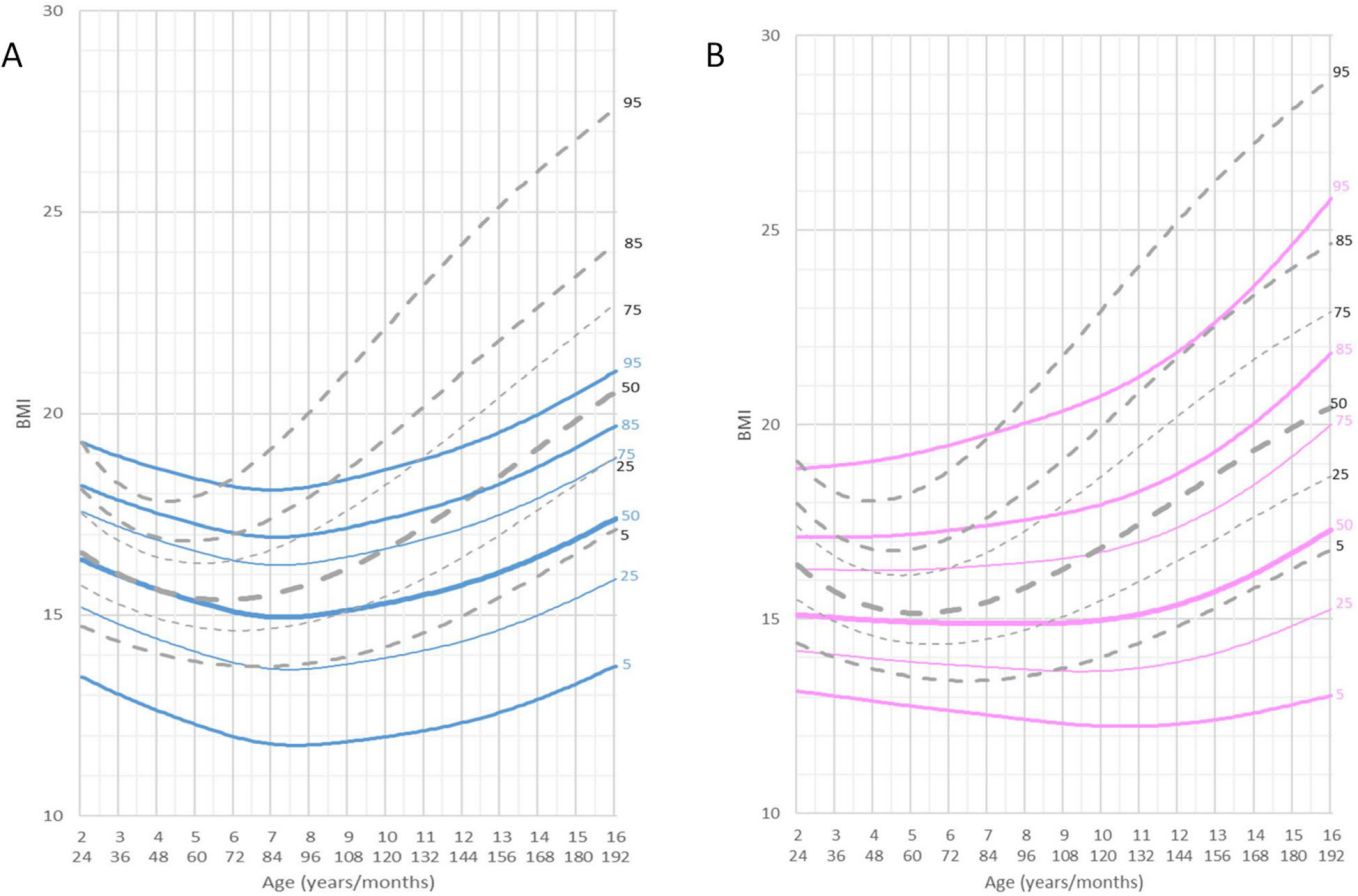
Constructed charts for the BMI (Kg/m^2^) for MWS patients in relation to the CDC reference charts (black) **a** for males (blue) **b** for females (pink)

BMI in the age range 0 to 6 years was slightly lower with respect to the normal reference group. Mean BMI for male patients at 6 years of age was 15 kg/m^2^ (mean reference group: 15.4 kg/m^2^) and for female patients was 14.9 kg/m^2^ (mean reference group: 15.3 kg/m^2^). Thereafter, between the ages of 6 and 16 years, the curve for BMI flattened out significantly in both genders, but in males the shift of the curve towards lower values was more pronounced and occurred earlier, at the age of 7 years, while in females it became significant after the age of 9 years (Fig. 5).

BMI of male patients aged 16 years (mean 17.2 kg/m^2^) was significantly lower compared to the reference group (mean 20.5 kg/m^2^). BMI of female patients aged 16 years (mean 17.1 kg/m^2^) was significantly lower compared to the reference group (mean 20.5 kg/m^2^).

### Head circumference

The mean number of head circumference measurements per patient in males was 6.54 (SD 4.79). For 39 patients we had at least 2 measurements, and for 31 patients we collected more than 4 measurements.

The mean number of head circumference measurements per patient in females was 8.54 (SD 6.23). For 51 patients we had at least 2 measurements, and for 41 patients we collected more than 4 measurements.

At birth, head circumference differed only slightly from the reference group in both genders. Indeed, during the first months of life, most measurements were between the 3rd and the 25th percentile of the curves for healthy controls. This changed after 1 year of age: MWS patients had a significantly smaller head circumference between the ages of 1 and 2 years. Between the ages of 2 and 3 years the curves flattened out even more and the head circumference was significantly smaller compared to healthy controls.

**Fig. 6:**
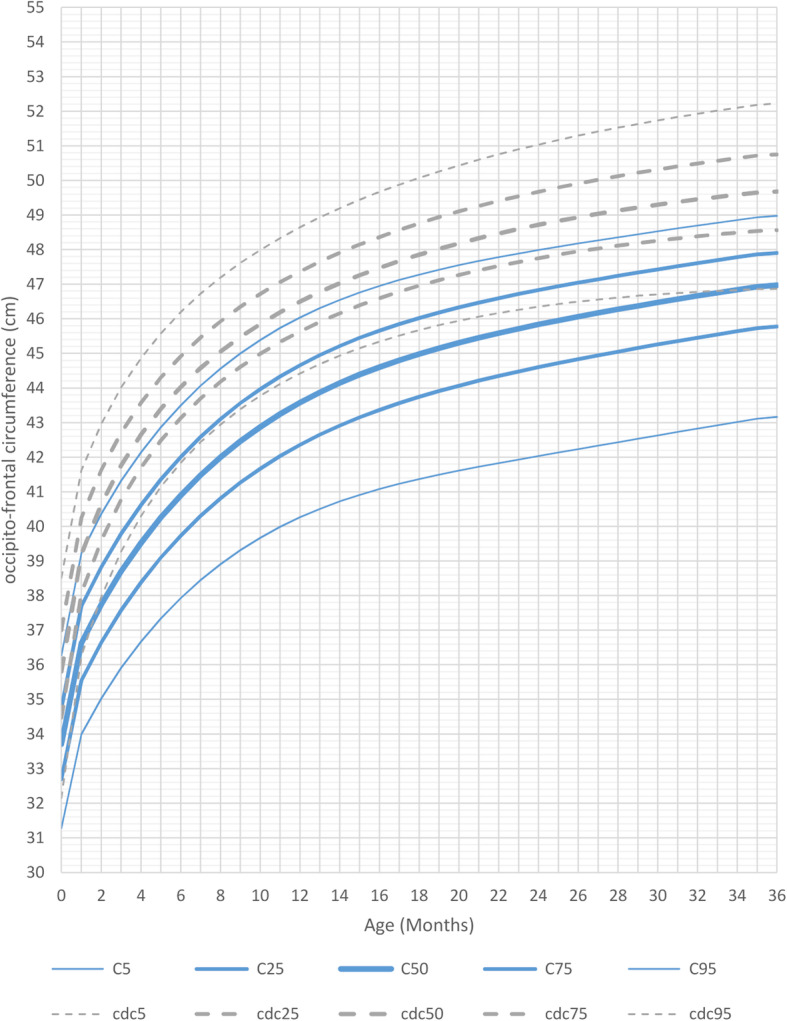
Constructed charts for the head circumference (cm) for MWS patients with age 0–3 years in relation to the CDC reference charts (black) for males (blue)

**Fig. 7:**
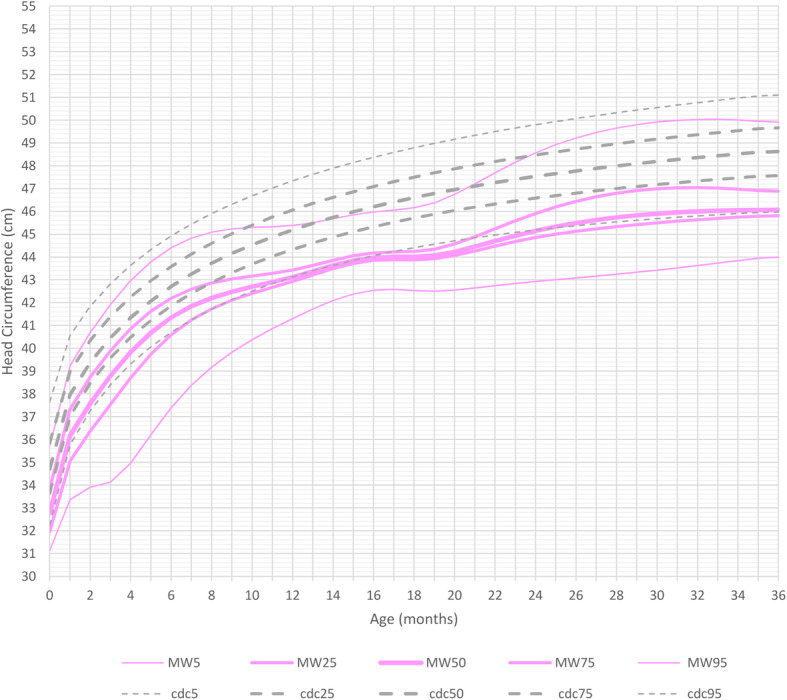
Constructed charts for the head circumference (cm) for MWS patients with age 0–3 years in relation to the CDC reference charts (black) for females (pink)

Mean head circumference for male patients at age 3 years was 47 cm (5th percentile, <− 2SD, mean reference group at 50th percentile: 50 cm) and for female patients was 46 cm (5th percentile, − 2SD, mean reference group at 50th percentile: 49 cm) (Figs. 6 and 7 and Additional file 7 Fig. RD5 Additional file 8 Fig.RD6).

It should be noted that not all children were microcephalic (at least 2 SD below the mean).

**Fig. 8:**
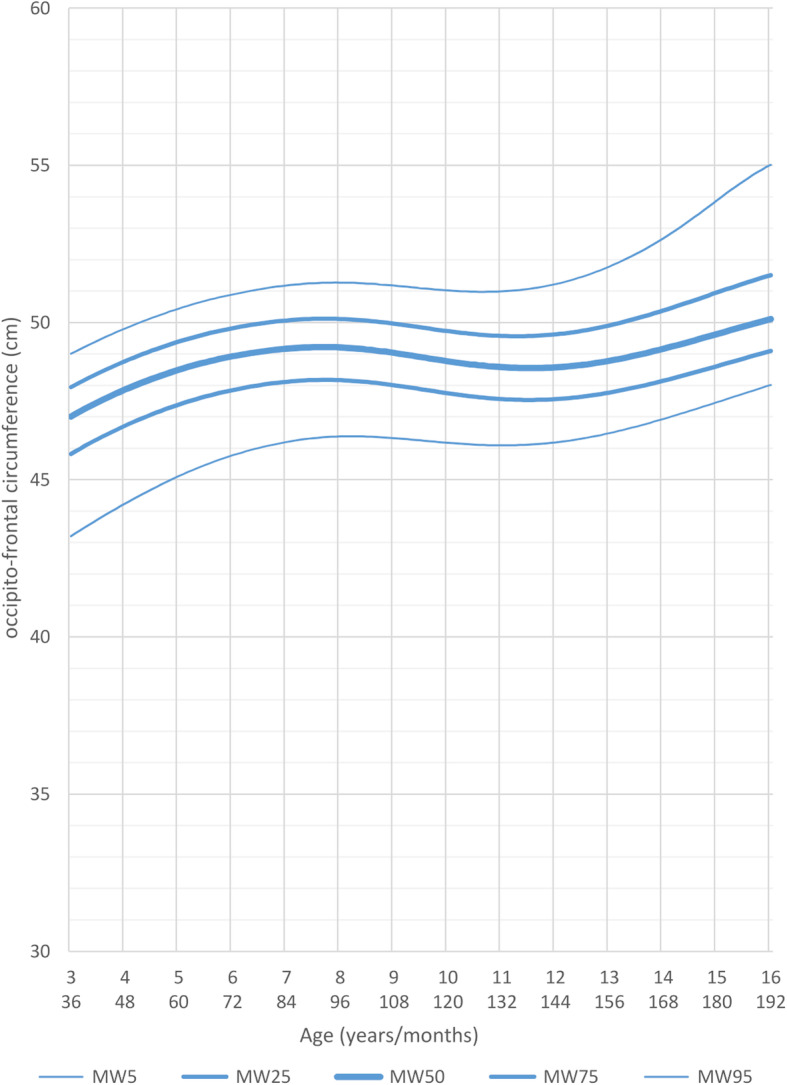
Constructed charts for the head circumference (cm) for MWS patients with age 3–16 years in relation to the Tanner reference charts (black) for males (blue)

**Fig. 9:**
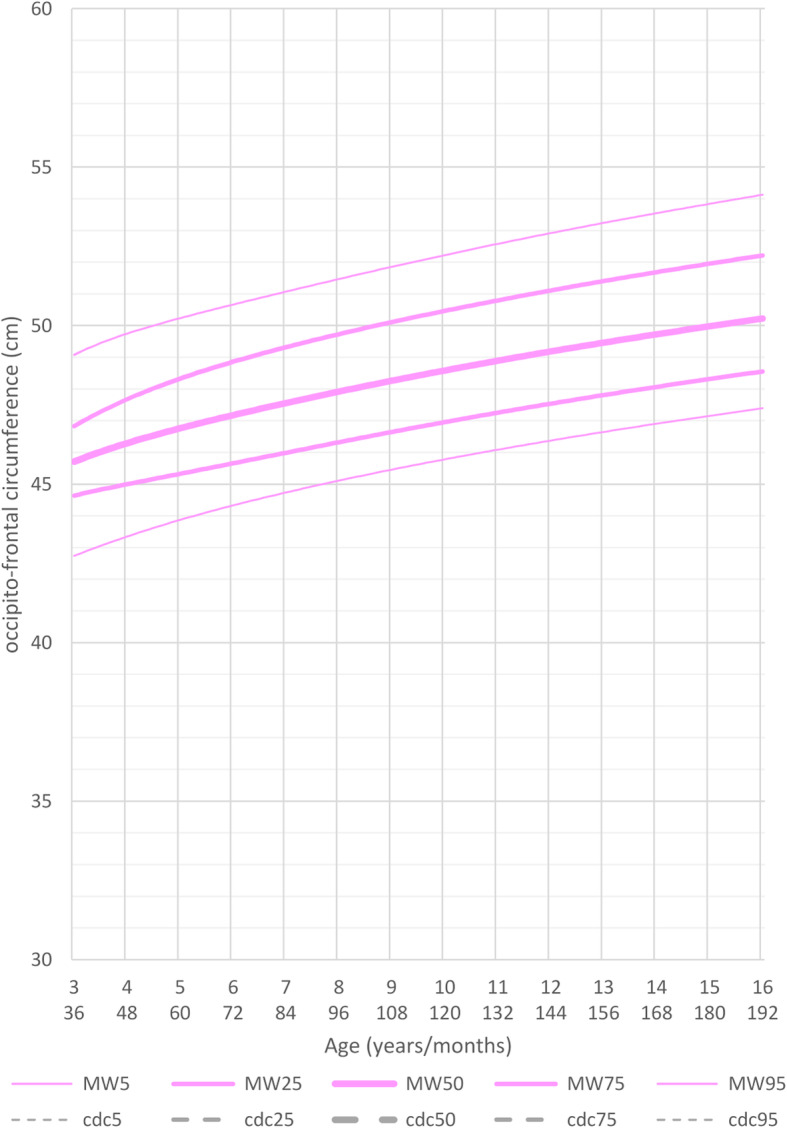
Constructed charts for the head circumference (cm) for MWS patients with age 3–16 years in relation to the Tanner reference charts (black) for females (pink)

The curves for males and females continued to flatten out in the following age ranges, up to the age of 16 years. Adolescent male patients at the age of 16 years had a mean head circumference of 52 cm (<3rd percentile, <−2 SD), the females had a mean head circumference of 49.8 cm (<3rd percentile, <−2SD) (Figs. 8 and 9).

## Discussion

Various genetic disorders are characterized by growth abnormalities. Specific growth charts developed for these conditions are useful for the clinical follow-up of these patients [10–20].

In this study, charts for height, weight, BMI and head circumference were created using a sample of 99 patients whose clinical diagnosis of MWS had been confirmed through genetic or cytogenetic testing.

Typically, children affected by MWS had normal length and weight at birth, and then showed a growth delay over time. The average head circumference was already slightly smaller than in the general population at birth (3rd-25th percentile) and showed a further delay during growth, leading to a mean head circumference in MWS patients below the 3rd centile of the general population. These results indicate that intrauterine growth retardation in MWS is not a consistent finding in terms of length and weight, and only plays a minor role in terms of head circumference.

In males, since the age of 2 months, the slope of the growth curves for length underwent an initial decrease. A significant further decrease was noted after 7 years of age, as the values for height became markedly distant from the reference curves of the general population. In females we observed a similar tendency, but the delay in the height growth chart took place considerably later, after 11 years of age.

Through these length, weight and head circumference growth charts we demonstrate that postnatal growth retardation in MWS becomes evident after the second month of life, is quite pronounced after 6 months and, after 1 year of age, growth flattens out even further. Males tend to be thinner than females and they also tend to have a similar head circumference when compared to females.

In addition, we observed the absence of the growth spurt during adolescence, as has already been observed in other genetic diseases, such as Turner syndrome and *SHOX-*related conditions [36, 37], but we do not yet have useful and sufficient data to calculate a final height, because the definition of MWS is quite recent and known adult individuals are still rare.

We also assessed BMI in MWS patients. BMI of male patients aged 16 years (mean 17.2 kg/m^2^) was found to be significantly lower when compared to the reference group (mean 20.5 kg/m^2^). BMI of female patients aged 16 years (mean 17.1 kg/m^2^) was significantly lower compared to the reference group (mean 20.5 kg/m^2^).

A limitation of the present study is the small absolute number of available subjects, which slightly reduces the precision of the growth curves, particularly in the adolescence age range. Nevertheless, this cohort includes a large portion of all well-described MWS patients in the world, and including only MWS patients with a confirmed *ZEB2* defect significantly increases the reliability of our results by excluding any misclassification of cases.

## Conclusions

The growth charts we prepared for height, weight, BMI and head circumference may be used to assess the growth of MWS children compared to the general population.

Specific growth charts can be useful for pediatricians and clinicians in evaluating growth-related issues and in providing optimal care to MWS individuals. Furthermore, they contribute to our understanding of the natural history of MWS.

The growth charts presented here may be put to good use all over the world, however they ought to be used carefully in MWS individuals with other ethnic backgrounds. In circumstances such as these, it is essential to consider variations according to the area of origin.

## Supplementary information

**Additional file 1: Table S1.** Number of measurements for male and female patients for height, weight and head circumference in relation to the age groups.

**Additional file 2: Table S2.** Model specification parameters by anthropometric measures and sex.

**Additional file 3: Figure S1.** RD1. Raw data of length and height (cm) for males showing the construction of the charts with individual data points.

**Additional file 4: Figure S2.** RD2. Raw data of length and height (cm) for females showing the construction of the charts with individual data points.

**Additional file 5: Figure S3.** RD3. Raw data of weight (Kg) for males showing the construction of the charts with individual data points.

**Additional file 6: Figure S4.** RD4. Raw data of weight (Kg) for females showing the construction of the charts with individual data points.

**Additional file 7: Figure S5.** RD5. Raw data of head circumference (cm) for males showing the construction of the charts with individual data points.

**Additional file 8: Figure S6.** RD6. Raw data of head circumference (cm) for females showing the construction of the charts with individual data points.

## Data Availability

The dataset is summarized in the additional files. The raw data used during the current study are available from the corresponding author upon reasonable request.

## Abbreviations

MWS: Mowat-Wilson syndrome
BMI: Body mass index
SD: Standard deviation
AIMW: Italian Mowat-Wilson syndrome association
