# Supplementary material for: Mowat-Wilson syndrome: growth charts
Source: Orphanet J Rare Dis. 2020 Jun 15;15:151. doi: 10.1186/s13023-020-01418-4 (PMC7294656; doi:10.1186/s13023-020-01418-4)
Supplement: Supplementary file 1 — Additional file 1: Table S1. Number of measurements for male and female patients for height, weight and head circumference in relation to the age groups. [file 13023_2020_1418_MOESM1_ESM.docx]

Additional file 1: Table 1 – Number of measurements for male and female patients for height, weight and head circumference in relation to the age groups.

| **Age** | **Length/Height** |  | **Weight** |  | **Head circumference** | |
| --- | --- | --- | --- | --- | --- | --- |
|  | **male** | **female** | **male** | **female** | **male** | **female** |
| at birth | 32 | 46 | 35 | 48 | 27 | 38 |
| after birth until < 1 year | 100 | 132 | 106 | 142 | 96 | 135 |
| 1 year until < 2 years | 60 | 75 | 63 | 83 | 45 | 67 |
| 2 years until < 3 years | 47 | 46 | 50 | 52 | 37 | 40 |
| 3 years until < 4 years | 28 | 35 | 30 | 38 | 19 | 27 |
| 4 years until < 5 years | 22 | 33 | 21 | 37 | 12 | 21 |
| 5 years until < 6 years | 18 | 27 | 20 | 32 | 9 | 21 |
| 6 years until < 7 years | 14 | 25 | 17 | 28 | 7 | 13 |
| 7 years until < 8 years | 15 | 24 | 15 | 30 | 5 | 14 |
| 8 years until < 9 years | 15 | 22 | 15 | 27 | 6 | 15 |
| 9 years until < 10 years | 15 | 20 | 17 | 21 | 5 | 15 |
| 10 years until < 11 years | 13 | 22 | 13 | 21 | 6 | 13 |
| 11 years until < 12 years | 11 | 18 | 12 | 18 | 3 | 6 |
| 12 years until < 13 years | 9 | 9 | 11 | 10 | 3 | 5 |
| 13 years until < 14 years | 5 | 6 | 7 | 8 | 3 | 2 |
| 14 years until < 15 years | 9 | 9 | 11 | 8 | 4 | 1 |
| 15 years until < 16 years | 5 | 11 | 6 | 12 | 3 | 3 |
| 16 years until < 17 years | 10 | 5 | 12 | 7 | 5 | 2 |
| 17 years until < 18 years | 5 | 4 | 8 | 5 | 1 | 2 |
| ≥ 18 years | 7 | 4 | 10 | 4 | 5 | 1 |
|  | 440 | 573 | 479 | 631 | 301 | 441 |
|  |  |  |  |  |  |  |
