# Supplementary material for: Mowat-Wilson syndrome: growth charts
Source: Orphanet J Rare Dis. 2020 Jun 15;15:151. doi: 10.1186/s13023-020-01418-4 (PMC7294656; doi:10.1186/s13023-020-01418-4)
Supplement: Supplementary file 2 — Additional file 2: Table S2. Model specification parameters by anthropometric measures and sex. [file 13023_2020_1418_MOESM2_ESM.docx]

| Y | Sex | Age (months) | Family | g(µ) | g(δ) | g(ν) | g(τ) | ξ | AIC | degree of freedom for h(µ/δ/ν/τ) |
| --- | --- | --- | --- | --- | --- | --- | --- | --- | --- | --- |
| Weight | M | ≤192 | BCTo, "Box-Cox-t-orig." | identity | log | identity | log | 0,836 | 2031,79 | 8/3/2/2 |
|  | F | ≤156 | BCPEo, "Box-Cox Power Exponential-orig." | identity | log | identity | log | 0,653 | 2430,31 | 9/2/2/3 |
| Length | M | ≤192 | BCCGo, "Box-Cox-Cole-Green-orig." | identity | log | identity |  | 0,752 | 2577,18 | 10/3/8/0 |
|  | F | ≤192 | BCPEo, "Box-Cox Power Exponential-orig." | identity | log | identity | log | 0,6805285 | 3398,45 | 10/2/5/5 |
| Head circumference | M | ≤192 | BCCGo, "Box-Cox-Cole-Green-orig." | identity | log | identity |  | 0,6357329 | 1.132,29 | 5/3/5/0 |
|  | F | ≤192 | BCPEo, "Box-Cox Power Exponential-orig." | identity | log | identity | log | 0,0540774 | 1767,03 | 6/2/3/5 |
| BMI | M | ≤192 | NO, "Normal" | identity | log |  |  | 1,008649 | 945,875 | 4/2/0/0 |
|  | F | ≤192 | BCCGo, "Box-Cox-Cole-Green-orig." | identity | log | identity |  | 1,499942 | 1374,13 | 4/2/2/0 |
|  |  |  |  |  |  |  |  |  |  |  |
|  | ξ=exponential parameter for age | | |  |  |  |  |  |  |  |
|  | g()=link functions | | |  |  |  |  |  |  |  |
|  | h()=non-parametric smoothing functions | | |  |  |  |  |  |  |  |
|  | AIC=Akaike Infromation Criterion  BMI=body mass index | | |  |  |  |  |  |  |  |

Additional file 2 Table 2: Model specification parameters by anthropometric measures and sex
