# Supplementary figures and images for: Mowat-Wilson syndrome: growth charts
Source: Orphanet J Rare Dis. 2020 Jun 15;15:151. doi: 10.1186/s13023-020-01418-4 (PMC7294656; doi:10.1186/s13023-020-01418-4)

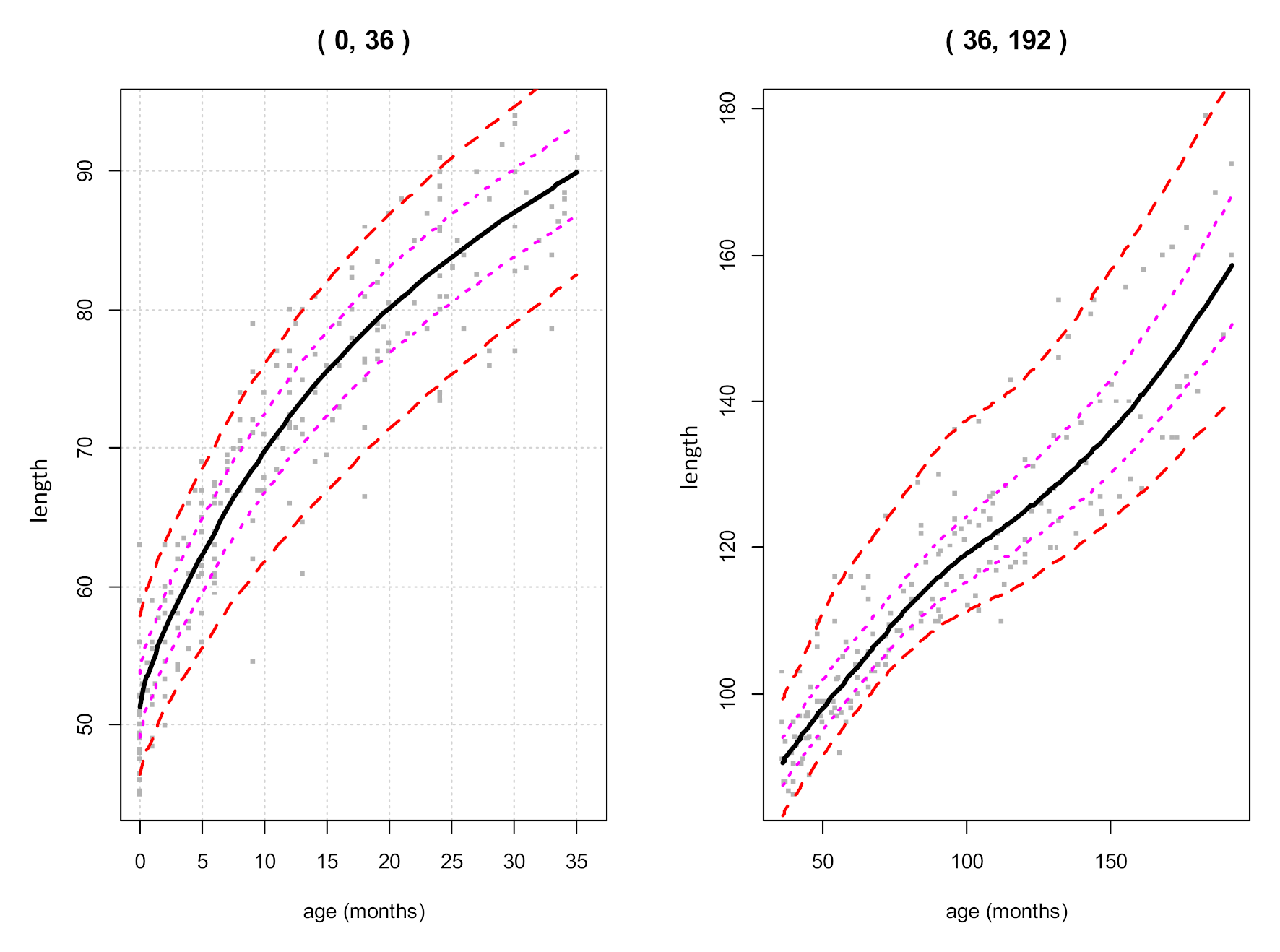

Supplement: Supplementary file 3 — Additional file 3: Figure S1. RD1. Raw data of length and height (cm) for males showing the construction of the charts with individual data points. [file 13023_2020_1418_MOESM3_ESM.tif]

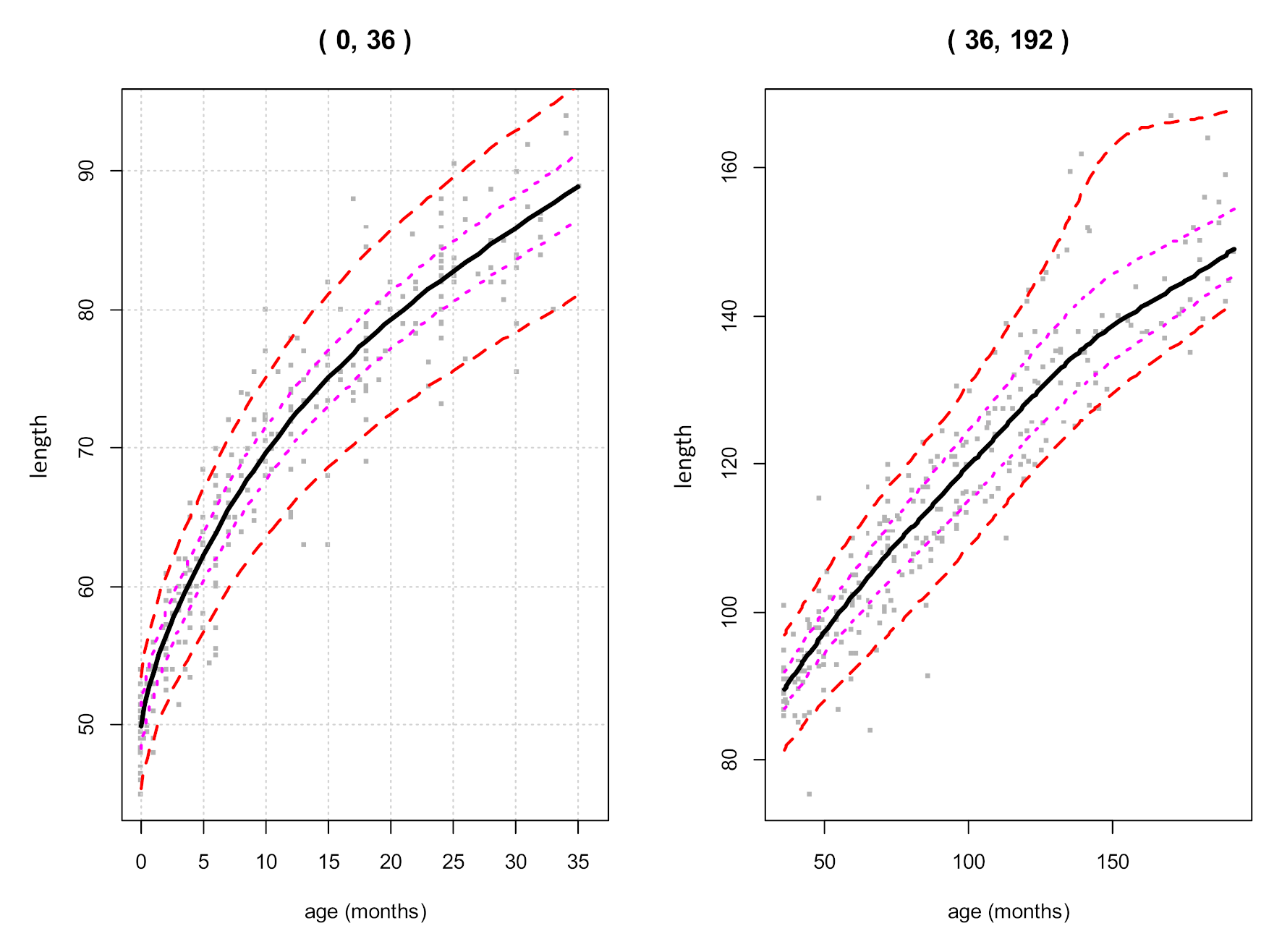

Supplement: Supplementary file 4 — Additional file 4: Figure S2. RD2. Raw data of length and height (cm) for females showing the construction of the charts with individual data points. [file 13023_2020_1418_MOESM4_ESM.tif]

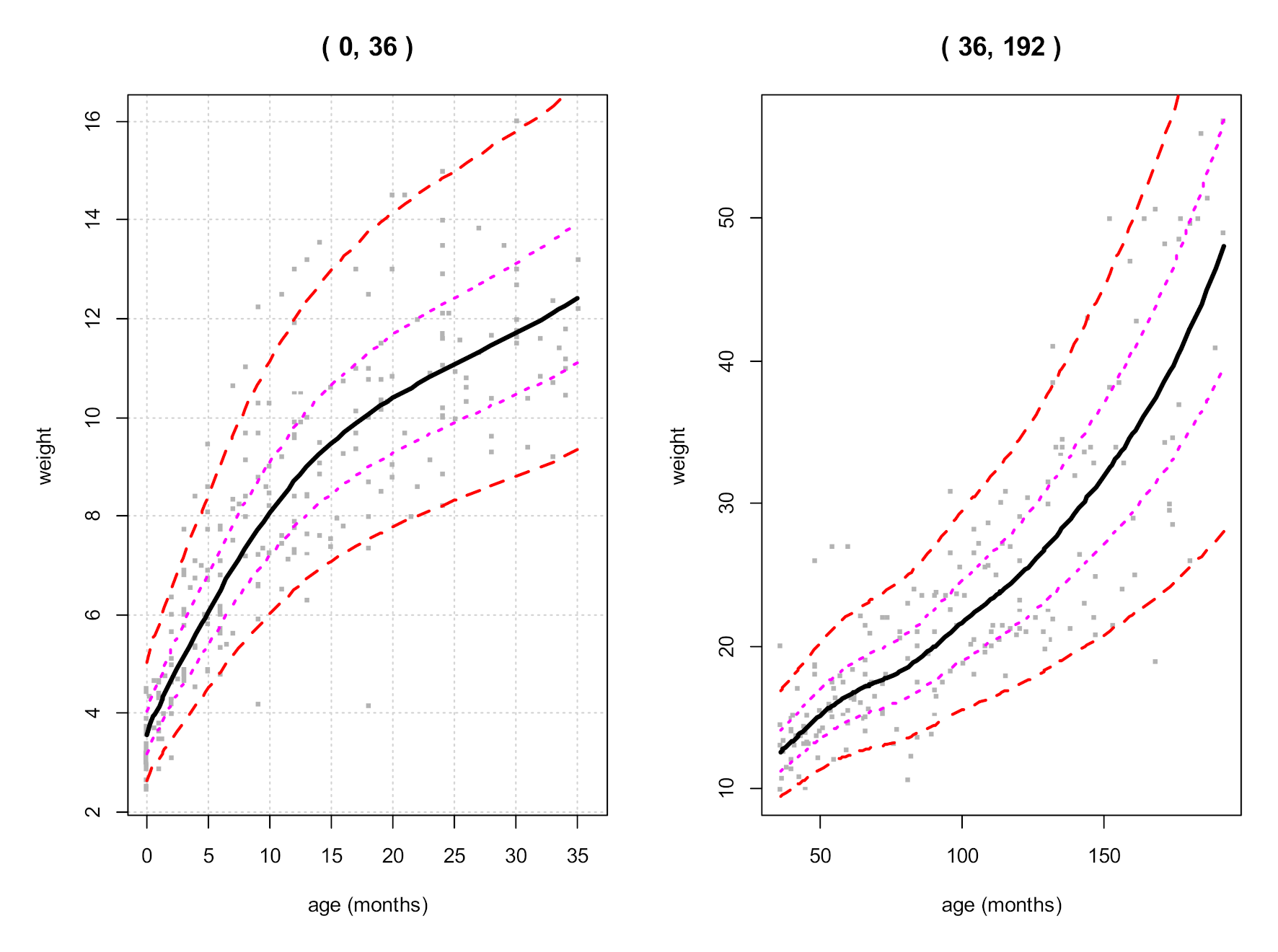

Supplement: Supplementary file 5 — Additional file 5: Figure S3. RD3. Raw data of weight (Kg) for males showing the construction of the charts with individual data points. [file 13023_2020_1418_MOESM5_ESM.tif]

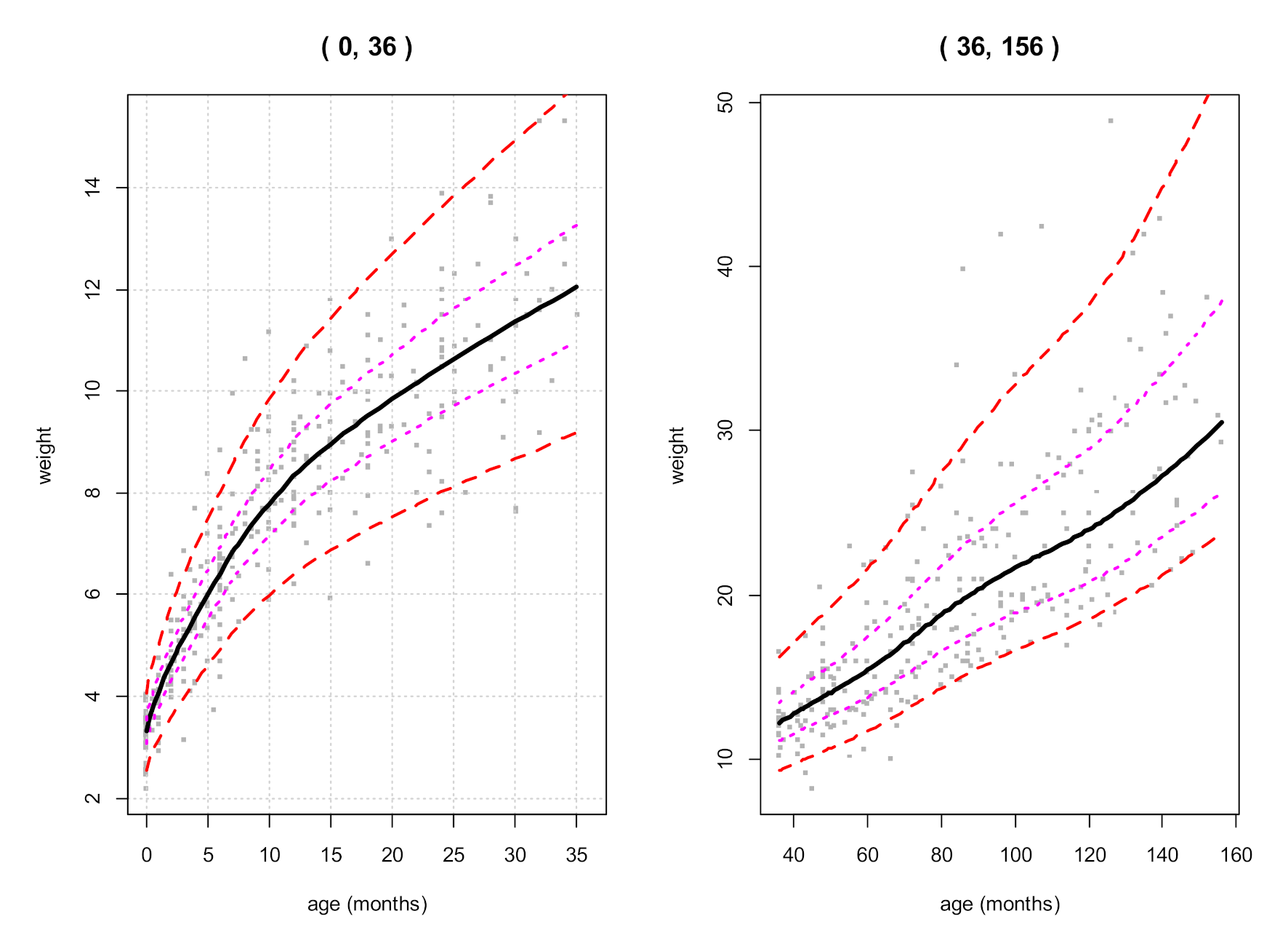

Supplement: Supplementary file 6 — Additional file 6: Figure S4. RD4. Raw data of weight (Kg) for females showing the construction of the charts with individual data points. [file 13023_2020_1418_MOESM6_ESM.tif]

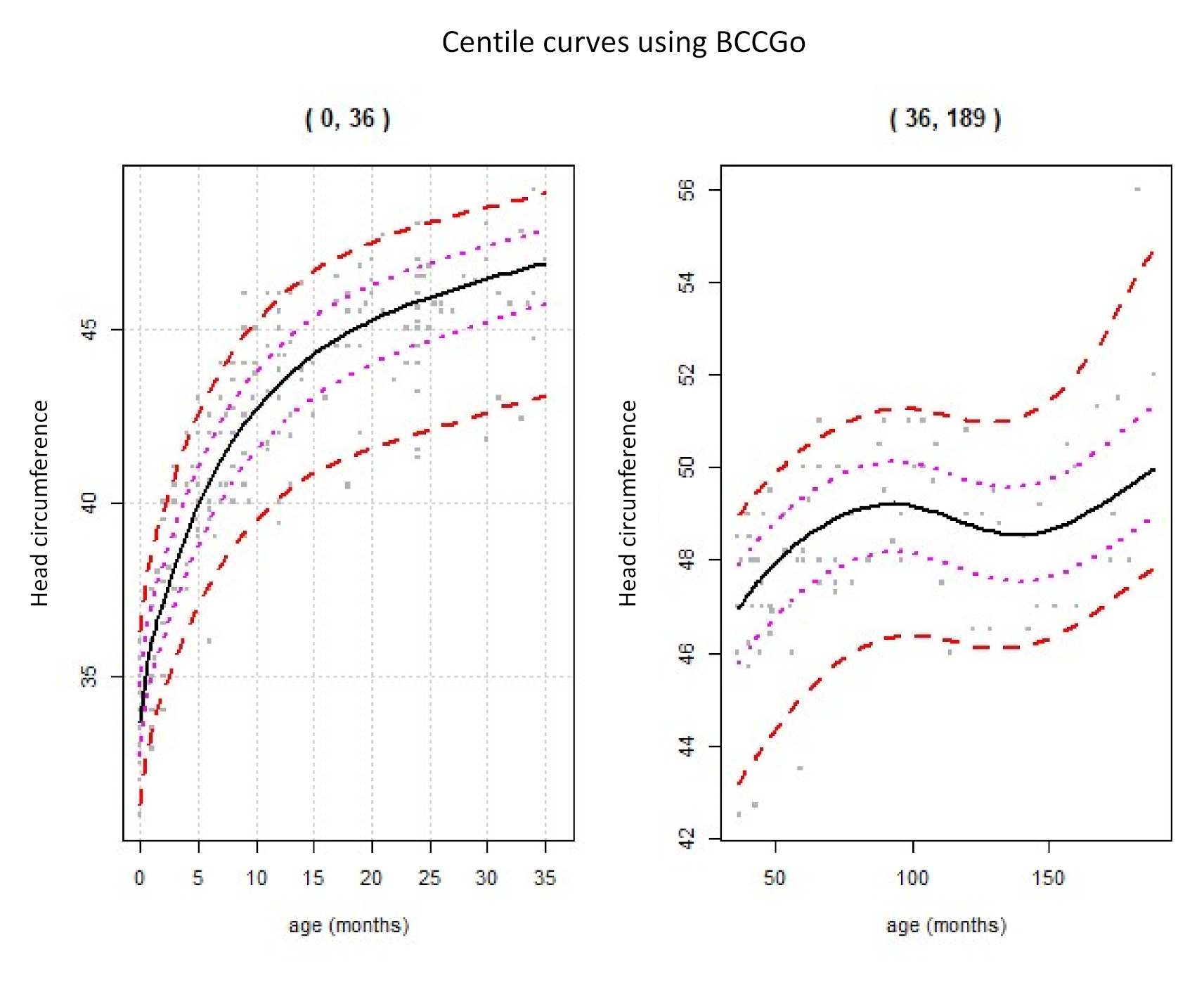

Supplement: Supplementary file 7 — Additional file 7: Figure S5. RD5. Raw data of head circumference (cm) for males showing the construction of the charts with individual data points. [file 13023_2020_1418_MOESM7_ESM.tif]

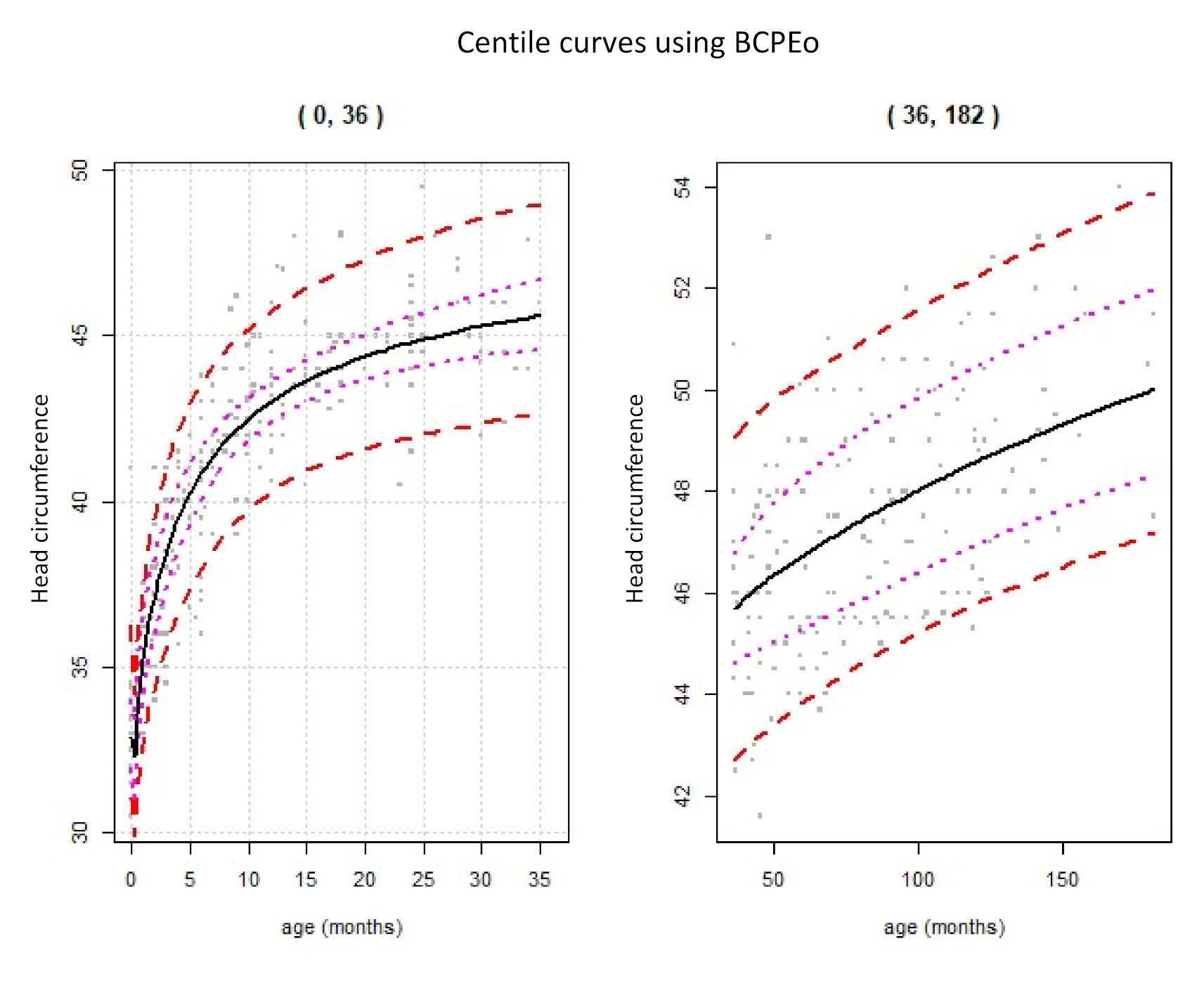

Supplement: Supplementary file 8 — Additional file 8: Figure S6. RD6. Raw data of head circumference (cm) for females showing the construction of the charts with individual data points. [file 13023_2020_1418_MOESM8_ESM.tif]
